# Evaluation of Relationship Between Sleep Bruxism and Headache Impact Test-6 (HIT-6) Scores: A Polysomnographic Study

**DOI:** 10.3389/fneur.2019.00487

**Published:** 2019-05-14

**Authors:** Helena Martynowicz, Joanna Smardz, Monika Michalek-Zrabkowska, Pawel Gac, Rafal Poreba, Anna Wojakowska, Grzegorz Mazur, Mieszko Wieckiewicz

**Affiliations:** ^1^Department of Internal Medicine, Occupational Diseases, Hypertension and Clinical Oncology, Wroclaw Medical University, Wroclaw, Poland; ^2^Department of Experimental Dentistry, Wroclaw Medical University, Wroclaw, Poland; ^3^Department of Hygiene, Wroclaw Medical University, Wroclaw, Poland

**Keywords:** sleep bruxism, headache, arousal, phasic bruxism, polysomnography

## Abstract

Sleep bruxism (SB) is a masticatory muscle activity during sleep characterized by teeth clenching or grinding and/or bracing or thrusting of the mandible. Morning headache is considered as a common symptom of SB; however, the relationship between SB and headache and its impact on patient's life is not clear. Therefore, the present study aimed to assess the relationship between SB using polysomnography with video/audio recording and Headache Impact Test-6 (HIT-6) scores. SB was evaluated in respondents by single-night diagnostic polysomnography with video/audio recording. The study found that Bruxism Episode Index was similar in the group with significant impact of headache on patient's life (HIT-6 score ≥ 50) and in group with little or no impact (HIT-6 score < 50). A statistically significant positive correlation was observed between bruxism associated with arousal and HIT-6 score (*r* = 0.51, *p* < 0.05) and between mixed bruxism and HIT-6 score (*r* = 0.58, *p* < 0.05) in the subgroup with phasic bruxism. The results indicated the relationship between SB and impact of severity of headache on the patient's life measured by HIT-6 is only modest. It was also found that the impact of severity of headache measured by HIT-6 is altered only in those with phasic bruxism and is associated with arousal. Further research should elucidate the factors influencing the relationship between SB and headache.

**Trial Registration:** Clinical Trials NCT03083405, WMU1/2017, https://clinicaltrials.gov/ct2/show/NCT03083405

## Introduction

Chronic headache defined as headache occurring on 15 or more days per month for at least 3 months is a major cause of pain and disability (1). The global prevalence of headache has been estimated to be as much as 42% (2). Thus, headache has an important socioeconomic impact and is considered as a burden for society. Migraine and tension-type headache are the most common types of primary headaches, while the most common secondary headaches are those attributed to head injury (1). Sleep and headache have a complex and extensive relationship (3). Sleep disturbances and stress are the most common headache triggers (4), and when these conditions coexist, they have an additive effect in patients with chronic headache (5). Insufficient sleep is prevalent among subjects with tension-type headache and is linked to exacerbation of symptoms (6). Poor sleep may contribute to increased sensitivity to pain, thus increasing the frequency of headache attacks. Around 15–74% of individuals with obstructive sleep apnea (OSA) suffer from morning headache (7), and morning headache are often unspecific.

SB is a masticatory muscle activity during sleep characterized by teeth clenching or grinding and/or bracing or thrusting of the mandible. ICD-10 classifies bruxism as a sleep disorder (G47.63). A recent international consensus defined SB as a masticatory muscle activity that occurs during sleep and is characterized as either rhythmic (phasic) or non-rhythmic (tonic) and suggested that SB should not be considered as a movement disorder or a sleep disorder in otherwise healthy individuals (8). The consensus also proposed that bruxism can be as graded “possible,” “probable,” and “definite.” “Possible bruxism” is diagnosed based on a questionnaire or an interview, “probable bruxism” is diagnosed based on self-report and a clinical examination, and “definite bruxism” is diagnosed based on polysomnography (PSG) with video/audio recording (9). The American Academy of Sleep Medicine (AASM) has classified SB as a sleep-related movement disorder in the International Classification of Sleep Disorders – Third Edition (ICSD-3) (10). Most SB episodes occur in the stages N1 and N2 of sleep (called non-rapid eye movement (non-REM) sleep). SB episodes commonly occur at sleep arousal (11), with accompanying increase in heart rate and motor activity (12, 13). SB can lead to tooth wear, tooth mobility, tongue/cheek indentation, masticatory muscle hypertrophy, pain in the temporomandibular joint, and masticatory muscles, and also headache (14).

Studies focusing on the association between bruxism and headaches have shown contradictory results; while some have concluded that there is an association between bruxism and headache (15, 16), one study did not support this assertion (17). Moreover, the methods used for evaluation in many of these studies were insufficient, e.g., SB was assessed using questionnaires or by clinical examination or only with the use of a portable electromyographic device (18). These studies also had a small sample size which was a common limitation (19). Therefore, the present study aimed to assess the relationship between SB using polysomnography with video/audio recording and Headache Impact Test-6 (HIT-6) scores.

## Materials and Methods

A total of 77 adult patients hospitalized for the assessment of SB in the sleep laboratory at the Department and Clinic of Internal Medicine, Occupational Diseases, Hypertension and Clinical Oncology of Wroclaw Medical University were included in the study. The patients were enrolled between March 2017 and April 2018 by qualified dentists in Clinic of Prosthetic Dentistry operating at the Department of Prosthetic Dentistry of the Wroclaw Medical University and other clinics dealing with comprehensive dental treatment. The diagnosis of probable sleep bruxism was made on positive clinical inspection of the following symptoms: masticatory muscle hypertrophy, indentations on the tongue or lip and/or a linear alba on the inner cheek, damage to the dental hard tissues (e.g., cracked teeth), mechanical wear of the teeth (i.e., attrition), or repetitive failures of restorative work/prosthodontic constructions and/or a positive self-report of teeth grinding, clenching, or bracing of the mandible during sleep (8).

The inclusion criteria considered were as follows: age over 18 years; diagnosis of probable SB on the basis of international consensus on the assessment of bruxism (8); absence of contraindications for PSG examination; and willingness to participate in the study. The exclusion criteria considered were as follows: severe systemic (including genetic) diseases; presence of secondary bruxism induced by systemic diseases, e.g., Parkinson's disease; use of medicines that can significantly affect the function of the nervous and muscular systems; severe mental illness and significant mental (including genetic) disorders; inability to undergo PSG, including severe mental impairment or Alzheimer's disease; presence of neurological disorders and/or neuropathic pain in the last six month; respiratory insufficiency, or active inflammation; treatment with or addicted to analgesic drugs and/or drugs that affect muscle and breath function; presence of active malignancy.

The headache impact test (HIT-6) was used to assess the impact of headache on the quality of life of the respondents. HIT-6 is a validated tool containing six questions on domains including social-role functioning, pain, emotional distress and well-being, cognitive functioning, and vitality (20). The HIT-6 scores 36–49 indicate that headache has no impact on the quality of life of the respondents, 50–55 indicate moderate impact, scores between 56 and 59 indicate a substantial impact, and scores ≥60 indicate a severe impact (21). For the purpose of this study, HIT-6 was translated into Polish by a native specialized in Medical English Terminology, and the questionnaire was double-checked by a physician specialized in the treatment of headaches.

For the assessment of SB, standard, multichannel, single-night diagnostic PSG was conducted using Nox-A1 (Nox Medical, Iceland) in the Sleep Laboratory at the Wroclaw Medical University. Polysomnograms were assessed in 30 s epochs according to the AASM standard criteria for sleep scoring (22). The following PSG outcome variables were assessed: sleep latency; total sleep time; sleep efficiency (%); and the percentages of N1, N2, N3, and REM sleep. Abnormal respiratory events were scored from the pressure airflow signal evaluated according to the standard criteria of the AASM Task Force (22). Electroencephalogram (frontal, central, and occipital regions), electrooculogram, electromyogram (submental), snoring, nasal pressure, rib cage, and abdominal movement by inductance plethysmography, heart rate, arterial oxygen saturation (SaO_2_) by finger pulse oximetry, activity, and body position were recorded. The mean, minimum and maximum pulse rate was measured by finger pulse oximetry. Apnea was defined as the absence of airflow for ≥10 s, and hypopnea was defined as a reduction in the amplitude of breathing by ≥30% for ≥10 s with a ≥3% decline in blood oxygen saturation or an arousal.

SB was assessed by electromyography (EMG) of bilateral masseter muscles and evaluation of video and audio recordings. Bruxism episodes were scored according to the AASM standards in the following three forms: phasic, tonic, and mixed. The AASM standards specify that for confirming SB, EMG activity had to be at least twice the amplitude of the background EMG and EMG bursts should not be separated by >3 s to be considered part of the same episode. A constant burst episode sustained over 2 s in masseter EMG recording was categorized as tonic, an episode including three or more bursts over 2 s was categorized as phasic, and a combination of tonic and phasic episode was categorized as mixed (23). The Bruxism Episode Index (BEI) measures the number of bruxism episodes per hour of sleep (<2: irrelevant SB; 2–4: mild/moderate SB; >4: severe SB) (24).

The scoring of SB episodes and analysis of collected data were performed by a qualified physician (H.M.) from the Sleep Laboratory at the Wroclaw Medical University.

Statistical analyses were conducted using Dell Statistical 13 software (Dell Inc., USA). The quantitative variables were expressed as arithmetic means and standard deviations, and the distribution of these variables was verified using the Shapiro–Wilk *W*-test. The qualitative variables were expressed as percentages, and *t*-test or the Mann–Whitney *U*-test was used for the evaluation of the independent quantitative variables in comparative analyses. The relationships between the analyzed variables were determined by correlation analyses. Those results with *p* < 0.05 were considered statistically significant.

This study was approved by the Ethical Committee of the Wroclaw Medical University (ID KB-195/2017). The written informed consents were obtained from the participants of this study.

## Results

The mean age of all the participants was 34.77 ± 10.86 years, and their mean Body Mass Index (BMI) was found to be 22.82 ± 3.89 kg/m^2^. In the studied group, women accounted for 72.7%, while men accounted for 27.3%.

HIT-6 subgroups did not differ in age and BMI. In the subgroup HIT-6 ≥ 50 compared to the HIT-6 < 50 subgroup, there were significantly more women (83.0 vs. 52.6%, *p* < 0.05). In contrast, the HIT-6 ≥ 60 and HIT-6 < 60 subgroups did not differ in sex.

The mean BEI of the participants was 4.42 ± 3.55. SB (BEI > 2) was diagnosed in 75.3% (*n* = 58), mild/moderate SB in 35.0% (*n* = 27), and severe SB in 40.2% (*n* = 31) of the participants.

The mean apnea–hypopnea index (AHI) of the participants was 5.02 ± 8.37. OSA (AHI >5) was diagnosed in 23.3% (*n* = 18) of the participants, of which mild (AHI 5–15), moderate (AHI 15–30), and severe OSA (AHI > 30) were diagnosed in 14% (*n* = 11), 5.19% (*n* = 4), and 3.8% (*n* = 3), respectively.

BEI was similar in the group with significant impact of headache on patient's life (HIT-6 score ≥ 50) and in group with little or no impact (HIT-6 score < 50) (Table 1). No statistically significant differences in respiratory and sleep indices were found between these studied groups (Table 2). In addition, no statistically significant correlation was found between HIT-6 score and BEI (*r* = −0.03, *p* > 0.05) and between HIT-6 score and AHI (*r* = −0.01, *p* > 0.05).

**Table 1:** The bruxism parameters in the group with headache (HIT-6 ≥ 50) and controls (HIT-6 < 50).

	**HIT-6 ≥ 50 (*n* = 53)**	**HIT-6 < 50 (*n* = 19)**	***p*-value**
BEI	4.37 ± 3.54	5.13 ± 3.69	0.43
BBI	5.20 ± 4.48	5.58 ± 6.49	0.65
apnea to bruxism index	0.88 ± 1.79	0.86 ± 1.73	0.97
arousal to bruxism index	2.14 ± 2.11	2.19 ± 2.05	0.92
Phasic bruxism index	1.43 ± 1.89	2.04 ± 2.59	0.29
Tonic bruxism index	1.89 ± 0.139	2.12 ± 1.75	0.58
Mixed bruxism index	1.10 ± 1.01	1.06 ± 0.97	0.86

*BEI, bruxism episode index; BBI, bruxism burst index; HIT-6, 6-item headache impact test score*.

**Table 2:** The respiratory and sleep parameters in in the group with headaches (HIT-6 ≥ 50) and controls (HIT-6 < 50).

	**HIT-6 > 50 (*n* = 53)**	**HIT-6 < 50 (*n* = 19)**	***p*-value**
AHI	5.03 ± 8.88	5.24 ± 8.00	0.93
ODI	4.38 ± 7.29	5.65 ± 8.43	0.53
Snore (%)	6.05 ± 13.29	10.52 ± 17.43	0.25
TST	416.65 ± 66.04	428.24 ± 40.39	0.48
SL	24.14 ± 23.20	22.24 ± 17.90	0.75
REML	111.59 ± 68.53	90.38 ± 35.63	0.20
WASO	36.31 ± 34.41	30.08 ± 24.68	0.47
SE	85.34 ± 11.39	87.24 ± 6.29	0.49
ArI	5.94 ± 4.52	4.69 ± 3.11	0.27
OAI	0.41 ± 2.02	0.23 ± 0.69	0.70
MAI	0.02 ± 0.10	0.01 ± 0.03	0.60
CAI	0.28 ± 0.51	0.20 ± 0.28	0.49
HI	3.76 ± 7.26	4.78 ± 7.46	0.60
C-S R	0.72 ± 2.02	0.79 ± 1.33	0.87
mean HR	62.79 ± 8.09	59.39 ± 8.06	0.13
max HR	91.23 ± 7.10	96.28 ± 5.59	0.56
min HR	49.30 ± 7.38	47.67 ± 6.00	0.40

*AHI, apnea–hypopnea index; ODI, oxygen desaturation index; TST, total sleep time; SL, sleep latency; REML, REM latency; WASO, wake after sleep onset; SE, sleep efficiency; ArI, Arausal index; OAI, obstructive apnea index; MAI, mixed apnea index; CAI, central apnea index; HI, hypopnea index; C-SR, Cheyne-Stokes respiration; HR, heart rate; HIT-6, 6-item headache impact test score*.

Furthermore, no statistically significant difference was observed in HIT-6 score between the group with SB (BEI >2) and the group without SB (BEI < 2) (55.52 ± 9.95 vs. 55.56 ± 8.30, *p* = 0.99). Similarly, no statistically significant difference was observed in HIT-6 score between the group with severe SB (BEI > 4) and the group with irrelevant or mild/moderate SB (BEI < 4) (54.13 ± 10.14 vs. 56.52 ± 9.03, *p* = 0.30) (Table 3).

**Table 3:** The HIT-6-score in bruxers (BEI > 2), non-bruxers (BEI < 2), severe bruxers (BEI > 4), and non-severe bruxers (BEI < 4).

**Group (*n*)**	**HIT-6 score**	***p*-value**
BEI >2 (*n* = 58)	55.52 ± 9.95	*p* = 0.99
BEI < 2 (*n* = 19)	55.56 ± 8.30	
BEI > 4 (*n* = 31)	54.13 ± 10.14	*p* = 0.30
BEI < 4 (*n* = 46)	56.52 ± 9.03	

*BEI, Bruxism Episode Index; HIT-6, 6-item headache impact test score*.

**Figure 1:**
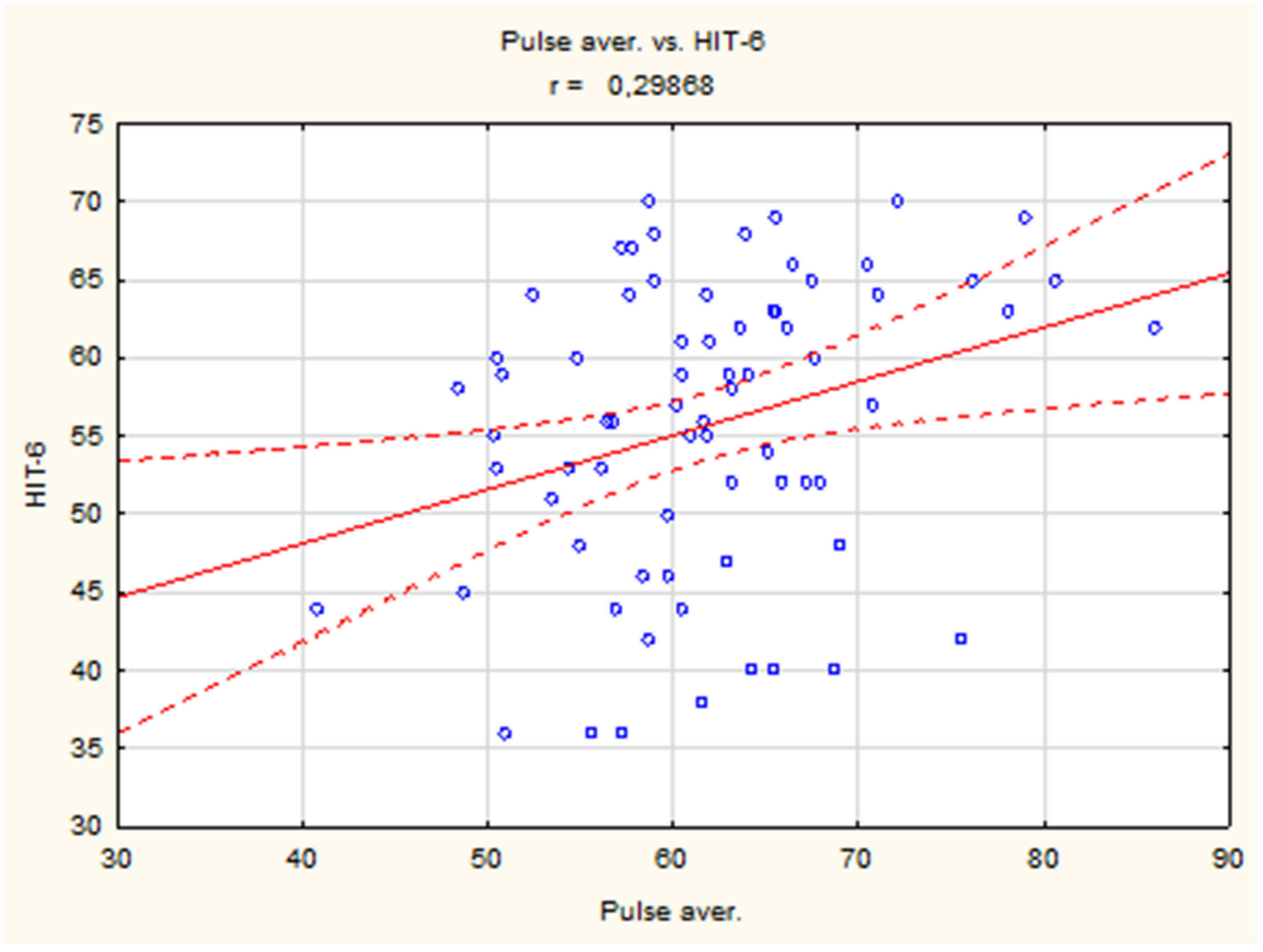
The correlation between mean heart rate and HIT-6 score in whole studied group.

The results showed a positive correlation between mean heart rate and HIT-6 score (*r* = 0.30, *p* < 0.05) (Figure 1), but there were no differences in mean, maximum, and minimum heart rate between the group with headache (HIT-6 score ≥ 50) and controls (HIT-6 score < 50).

There was no statistically significant difference between pulse rate in the group with significant impact of headache on patient's life (HIT-6 score ≥ 50) and in group with little or no impact (HIT-6 score < 50). However, we compared groups with severe impact of headache on the ability of subjects to function at work, school, home, and in social situations measured by HIT-6 (HIT-6 score ≥ 60) and controls (HIT-score < 60) due to fact that severe headache may increase pulse rate. In the group with severe impact of headache (HIT-6 score ≥ 60), the mean heart rate and minimum heart rate were higher compared to the control group (HIT-6 score < 60) (Table 4).

**Table 4:** The heart rate in the group with headache (HIT-6 score ≥ 50), (HIT-6 score ≥ 60) and in controls.

	**HIT-6 ≥ 50**	**HIT-6 < 50**	***p*-value**	**HIT- 6 ≥ 60**	**HIT- 6 < 60**	***p*-value**
Mean HR	62.79 ± 8.09	59.39 ± 0.06	0.13	65.47 ± 8.54	59.34 ± 6.88	0.00^*^
Max HR	98.83 ± 17.42	96.28 ± 5.59	0.55	102.03 ± 20.87	95.28 ± 8.31	0.07
Min HR	49.30 ± 7.38	47.67 ± 6.00	0.40	51.23 ± 7.84	47.14 ± 5.95	0.02^*^

*Mean HR, mean heart rate; max HR, maximum heart rate; min HR, minimum heart rate; HIT-6, 6-item headache impact test score*,

* *p < 0.05*.

**Figure 2:**
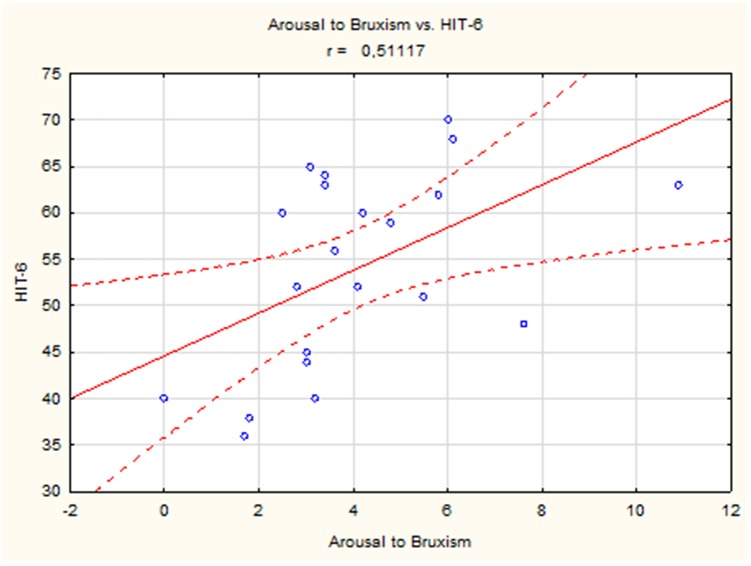
The correlation between bruxism associated with arousal and HIT-6 score in group with phasic bruxism>2.

**Figure 3:**
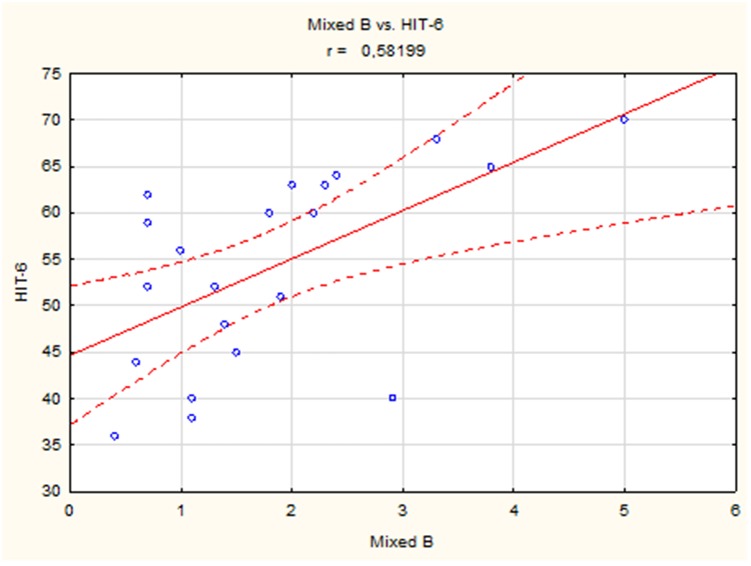
The correlation between mixed bruxism and HIT-6 score in group with phasic bruxism>2.

A positive linear correlation was observed between sleep bruxism associated with arousal and HIT-6 score (*r* = 0.51, *p* < 0.05) (Figure 2) and between mixed bruxism and HIT-6 score (*r* = 0.58, *p* < 0.05) (Figure 3) in the subgroup with phasic bruxism. In contrast, no statistically significant correlation was found between bruxism parameters and HIT-6 score in the subgroup with tonic bruxism.

## Discussion

All participants of the study were diagnosed with SB based on a positive physical examination and/or a positive interview (probable bruxism) (8). However, SB was confirmed only in 75% of the participants who underwent PSG with audio/video recording, and the diagnosis of SB in the remaining 25% of patients was false positive. Thus, it is worth noting that PSG with audio/video recording can serve as a gold standard for the diagnosis of SB.

HIT-6 score was higher than 49 points in bruxers (BEI > 2) and non-bruxers (BEI < 2), suggesting the impact of headaches on the ability of subjects to function at work, school, home, and in social situations in both the groups. The most interesting finding of this study was the absence of significant difference in HIT-6 score between bruxers (BEI > 2) and non-bruxers (BEI < 2) (Table 4). SB-related headache is described as a tension-type headache occurring in the morning or during the day (24); however, there is little evidence of any relationship between headache and bruxism (15, 16, 25). This is consistent with the findings of some case reports (26–28). Recently, a PSG study showed that SB may modestly exacerbate headache both in patients with mild brain injury and healthy controls; however, the number of bruxers included in the study was small (29). Recently, a study showed that there was no significant correlation between self-reported SB and the intensity of pain associated with temporomandibular disorders (TMDs) (30). Porporatti et al. also did not find any association between self-reported bruxism and primary headache (31) in their study. Similarly, no relationship was found between the frequency of SB diagnosed using a miniature disposable device and the prevalence of headache in an adolescent population (32). However, it was previously described that sleep bruxers with low frequencies of orofacial activities were at more risk of reporting pain (16).

SB may be associated with TMDs, and headache is often an accompanying symptom (33). Wagner et al. have showed an association between headache and TMDs (*p* < 0.001) and between headache and anxiety (*p* = 0.002), but not between headache and bruxism (*p* = 0.670) (17). Recently, it was showed that the presence of SB did not increase the risk for any type of headache, but SB coexisting with painful TMDs greatly increased the risk for episodic migraine, episodic tension-type headache, and especially for chronic migraine (34). In this study, the association between pain, bruxism, and TMDs was not investigated, and thus, the influence of TMDs on pain cannot be excluded in bruxers.

OSA was diagnosed in only 23% of the study group, and most of the cases (14 out of 18) had just mild OSA. Therefore, the impact of OSA as a compounding factor was inconsiderable.

Thus, the available scientific literature does not support the view that bruxism is a cause of pain (35, 36). This study also found no association between impact of headache on the ability of subjects to function at work, school, home, and in social situations measured by HIT-6 and severity of bruxism. However, a significant correlation was found between mixed bruxism and HIT-6 score in the subgroup with phasic bruxism. This result indicates a very modest relationship, especially for phasic bruxism. Interestingly, if bruxism was accompanied by arousal, a significant correlation with HIT-6 score was observed. This correlation may indicate the role of arousal in the etiology of headache severity impact on life in bruxers. This observation is in agreement with a previously reported association of worse sleep quality and higher intensity of headache (37).

A positive correlation between mean heart rate and HIT-6 score was observed in the study. Moreover, the mean heart rate was higher in the group with severe impact of headache on the ability of subjects to function at work, school, home, and in social situations measured by HIT-6 (HIT-6 score > 60) compared to controls (HIT-6 score < 60) (Table 4). The increased heart rate may be caused by pain in bruxers, but other factors may also be involved, e.g., increased sympathetic drive (38), inflammation, stress, anxiety (39), and stimulants like alcohol (40, 41) or caffeine (42). However, these factors were not investigated in this study.

This study has a few limitations. First of all, the clinical diagnosis of headache has not been made and the other possible causes of headache in bruxers have not been investigated. The classification of the reported headache according to the ICHD-3 has been not considered. The results for relevant subgroups could have been obfuscated. It has been showed that migraine and frequency of headache are associated with painful TMD in adolescents (43). There may be a central working mechanism overlapping TMD and headache (44). The further studies on association between bruxism, headache and TMD regarding different types of headache are needed.

## Conclusion

The study showed the relationship between sleep bruxism and impact of severity of headache on the patient's life measured by HIT-6 is only modest. It was also found that the impact of severity of headache measured by HIT-6 is altered only in those with phasic bruxism and is associated with arousal. Further research should elucidate the factors influencing the relationship between SB and headache.

## Ethics Statement

This study was approved by the Ethical Committee of the Wroclaw Medical University (ID KB-195/2017). Patients were required to sign a consent form for participating in the study.

## Author Contributions

HM created the research concept, analyzed the data, and wrote the manuscript. JS and MM-Z recruited patients for the study and collected data. RP and PG performed the statistical analysis. AW collected the references. GM revised the manuscript before submission. MW created the research concept, recruited patients for the study, evaluated the content, edited the manuscript, and finally revised it before submission. All authors read and approved the final manuscript.

### Conflict of Interest Statement

The authors declare that the research was conducted in the absence of any commercial or financial relationships that could be construed as a potential conflict of interest.
